# Clinical Characteristics of and Risk Factors for Chronic Kidney Disease Among Adults and Children: An Analysis of the CURE-CKD Registry

**DOI:** 10.1001/jamanetworkopen.2019.18169

**Published:** 2019-12-20

**Authors:** Katherine R. Tuttle, Radica Z. Alicic, O. Kenrik Duru, Cami R. Jones, Kenn B. Daratha, Susanne B. Nicholas, Sterling M. McPherson, Joshua J. Neumiller, Douglas S. Bell, Carol M. Mangione, Keith C. Norris

**Affiliations:** 1 Providence St Joseph Health, Providence Medical Research Center, Spokane, Washington; 2 University of Washington School of Medicine, Seattle; 3 Division of General Internal Medicine, Department of Medicine, David Geffen School of Medicine, University of California, Los Angeles; 4 Division of Nephrology, University of California, Los Angeles; 5 Elson S. Floyd College of Medicine, Washington State University, Spokane; 6 College of Pharmacy and Pharmaceutical Sciences, Washington State University, Spokane

## Abstract

**Importance:**

Chronic kidney disease (CKD) is serious and common, yet recognition and public health responses are limited.

**Objective:**

To describe clinical features of, prevalence of, major risk factors for, and care for CKD among patients treated in 2 large US health care systems.

**Design, Setting, and Participants:**

This cohort study collected data from the Center for Kidney Disease Research, Education, and Hope (CURE-CKD) registry, an electronic health record–based registry jointly curated and sponsored by Providence St Joseph Health and the University of California, Los Angeles. Patients were adults and children with CKD (excluding end-stage kidney disease) and adults at risk of CKD (ie, with diabetes, hypertension, or prediabetes) identified by laboratory values, vital signs, prescriptions, and administrative codes. Data were collected from January 2006 through December 2017, with analyses performed from March 2019 through November 2019.

**Exposures:**

Diabetes, hypertension, and prediabetes.

**Main Outcomes and Measures:**

Clinical and demographic characteristics, prevalence, and prescribed medications.

**Results:**

Of 2 625 963 adults and children in the sample, 606 064 adults (23.1%) with CKD had a median (interquartile range [IQR]) age of 70 (59-81) years, with 338 785 women (55.9%) and 434 474 non-Latino white individuals (71.7%). A total of 12 591 children (0.4%) with CKD had a median (IQR) age of 6 (1-13) years, with 7079 girls (56.2%) and 6653 non-Latino white children (52.8%). Median (IQR) estimated glomerular filtration rate was 53 (41-61) mL/min/1.73 m^2^ among adults and 70 (50-95) mL/min/1.73 m^2^ in children. Prevalence rates for CKD in adults were 4.8% overall (606 064 of 12 669 700) with 1.6% (93 644 of 6 011 129) during 2006 to 2009, 5.7% (393 455 of 6 903 084) during 2010 to 2013, and 8.4% (683 574 of 8 179 860) during 2014 to 2017 (*P* < .001). A total of 226 693 patients (37.4%) had category 3a CKD; 100 239 (16.5%), category 3b CKD; 39 125 (6.5%), category 4 CKD; and 20 328 (3.4%), category 5 CKD. Among adults with CKD, albuminuria and proteinuria assessments were available in 52 551 (8.7%) and 25 035 (4.1%) patients, respectively. A renin-angiotensin system inhibitor was prescribed to 124 575 patients (20.6%), and 204 307 (33.7%) received nonsteroidal anti-inflammatory drugs or proton pump inhibitors. Of 1 973 258 adults (75.1%) at risk, one-quarter had diabetes or prediabetes (512 299 [26.0%]), nearly half had hypertension (955 812 [48.4%]), and one-quarter had both hypertension and diabetes or prediabetes (505 147 [25.6%]).

**Conclusions and Relevance:**

This registry-based cohort study revealed a burgeoning number of patients with CKD and its major risk factors. Rates of identification and use of kidney protective agents were low, while potential nephrotoxin use was widespread, underscoring the pressing need for practice-based improvements in CKD prevention, recognition, and treatment.

## Key Points

**Question:**

What are the clinical characteristics of and major risk factors for chronic kidney disease among patients in 2 large US health care systems?

**Findings:**

In this cohort study of the Center for Kidney Research, Education, and Hope (CURE-CKD) registry, more than 2.6 million adults and children had chronic kidney disease or were at risk. Albuminuria or proteinuria was tested in approximately one-eighth of adults with chronic kidney disease, renin-angiotensin system inhibitors were prescribed to one-fifth, and nonsteroidal anti-inflammatory agents or proton pump inhibitors were prescribed to more than one-third.

**Meaning:**

Despite common occurrence of chronic kidney disease, rates of identification and use of kidney protective agents were low, while use of potential nephrotoxins was widespread.

## Teaser

This cohort study examines the clinical features of, prevalence of, major risk factors for, and care for chronic kidney disease among adults and children treated in 2 large US health care systems.

## Introduction

Chronic kidney disease (CKD) is a serious and common disease, and it eventuates in multiple complications, including premature mortality and end-stage kidney disease (ESKD).^1,2,3^ An estimated 1 in 7 to 10 adults worldwide have CKD, with only approximately 10% surviving to ESKD and only half of survivors receiving dialysis or a kidney transplant because of lack of access or high costs.^3^ From 1990 to 2016, the prevalence of CKD increased by 90%, and related deaths, mainly due to cardiovascular diseases and infections, nearly doubled in the United States and globally.^4,5,6^ In high-income countries, 2% to 3% of annual health care costs are devoted to the 0.03% of the population with ESKD.^7^

The increasing prevalence of CKD is closely tied to the increase of at-risk populations with diabetes, hypertension, and prediabetes. Indeed, diabetes is the leading cause of CKD and a global health emergency, with 425 million individuals affected worldwide in 2017 and a projected 629 million individuals affected by 2045.^8,9,10^ Hypertension is the second most frequent cause of CKD, affecting nearly one-third of US adults and 1.13 billion people globally in 2015.^11,12^ The estimated population size for prediabetes was 78.5 million among adults in the United States between 2011 and 2014, and nearly one-tenth have been reported with CKD.^13^ Even so, awareness of CKD and its major risk factors remains strikingly low among health care professionals and patients alike.^14,15,16^

The Advancing American Kidney Health initiative was recently launched by a US executive order calling for new approaches to prevent and treat CKD, with a goal of reducing ESKD incidence 25% by 2030.^17^ Real-world data from electronic health records (EHRs) provide an ideal platform to answer this call by improving CKD detection, tracking, and public health responses. The Center for Kidney Disease Research, Education, and Hope (CURE-CKD) registry contains detailed patient-level EHR data from more than 2.6 million adults and children with CKD and at risk of CKD during 12 inclusive years.^18^ The objective of this study was to describe baseline clinical features of, prevalence of, major risk factors for, and care for CKD based on data from the CURE-CKD registry.

## Methods

The study was approved by Providence St Joseph Health (PSJH; Washington, Montana, Oregon, Alaska, and California) and the University of California, Los Angeles (UCLA; California) Health institutional review boards, which determined that written informed consent was not required for this limited data set. Data use agreements between PSJH and UCLA Health formed the framework for data sharing, stewardship, and security. This study was conducted according to the Strengthening the Reporting of Observational Studies in Epidemiology (STROBE) reporting guideline for cohort studies.^19^

### Study Design

Detailed methodology for CURE-CKD has been previously published.^18^ The formation of CURE-CKD was supported by institutional funding from PSJH and UCLA Health. Both health care systems use Epic EHRs (Epic Systems). The first phase of CURE-CKD created a data repository with patient information from EHRs with at least 1 measure indicating CKD, diabetes, prediabetes, or hypertension based on patient-level laboratory values, vital signs, prescription medications, and administrative codes from January 1, 2006, to December 31, 2017. Electronic health record data for these patients were extracted from ambulatory and inpatient encounters. Unstructured data from the EHRs were not extracted. The total number of patients with encounters and serum creatinine measures for the health care systems was also recorded. Repository updates are performed annually.

The second phase crafted an EHR-based registry of participants with CKD and at risk for CKD derived from the repository. The first 90 days a patient was included in the registry were considered the baseline period. Registry criteria were based on established clinical practice guidelines for CKD (eTable 1 in the Supplement). Adults (ie, aged ≥18 years) were included with 2 or more of the following laboratory measurements recorded at least 90 days apart: estimated glomerular filtration rate (eGFR) less than 60 mL/min/1.73 m^2^, calculated from serum creatinine levels using the Chronic Kidney Disease Epidemiology equation; urine albumin to creatinine ratio greater than 30 mg/g; and urine protein to creatinine ratio greater than 150 mg/g.^20,21^ Children (ie, aged <18 years) with CKD were identified using the same criteria, except the bedside Schwartz equation was used to calculate eGFR from serum creatinine levels.^22^ We identified CKD categories 1 and 2 by an administrative code, urine albumin to creatinine ratio greater than 30 mg/g, and/or urine protein to creatinine ratio greater than 150 mg/g. We identified CKD categories 3 to 5 based on eGFR and/or administrative code. Patients with ESKD treated with dialysis or kidney transplant were excluded. Participants with diabetes, prediabetes, and hypertension were identified by clinical practice guidelines and published criteria for EHR identification^23,24,25^ (eTable 1 in the Supplement).

### Statistical Analysis

Data analyses were performed from March 2019 through November 2019. Continuous variables are reported as mean and SD or as median and interquartile range (IQR) for skewed or kurtotic distributions. Categorical variables are reported as frequencies and percentages. The Pearson χ^2^ test for independence was used to determine differences between categorical variables. Prevalence rates for CKD among adults are presented as a combined data set from PSJH and UCLA Health and by each system. To address sources of bias in CKD prevalence rates, data were analyzed as proportions based on the 3 following definitions for CKD: (1) CURE-CKD entry criteria, (2) 2 measurements of eGFR less than 60 mL/min/1.73 m^2^ at least 90 days apart; and (3) 1 measurement of eGFR less than 60 mL/min/1.73 m^2^. Serial prevalence rates of CKD overall, by categories, and prescription medication use during 3 periods (ie, 2006-2009, 2010-2013, and 2014-2017) were analyzed by logistic regression models. Prevalence was adjusted for age, sex, and race/ethnicity in the models using repository data (ie, CKD by 1 or 2 eGFR measurements). Adjustments could not be made for CKD prevalence with the denominator based on the total number of patients with encounters because the institutional review board approvals did not include data extraction for age, sex, and race/ethnicity from the total populations in the health care system.

To reduce risk of type I error, a 2-tailed *P* < .001 was the a priori threshold for statistical significance because of the large sample size and resultant high level of statistical power. Because overall CKD participant characteristics, except distribution of geolocation, were similar between PSJH and UCLA Health, findings other than prevalence are presented from a jointly curated data set. Descriptive statistics and the Pearson χ^2^ test were conducted with SQL Server Management Studio 2012 version 11.0.2100.60 (Microsoft Corp); tests for normality and logistic regression were completed using SPSS statistical software version 23 (IBM Corp).

## Results

### Demographic Characteristics in Adults and Children With CKD and Adults At Risk of CKD

A total of 2 625 963 adults and children were included in the sample. The cohort of adults with CKD included 606 064 individuals (23.1%), including 338 785 women (55.9%), 434 474 non-Latino white individuals (71.7%), 17 625 Latino individuals (2.9%), 29 974 black individuals (4.9%), 32 850 Asian individuals (5.4%), 5461 American Indian and Alaska Native individuals (0.9%), and 3899 Hawaiian and Pacific Islander individuals (0.6%) (Table 1). The median (IQR) age among adults was 70 (59-81) years. The proportions with CKD were highest among those aged 60 to 89 years (401 541 [66.3%]). A total of 12 591 children (0.4%) with CKD included 7079 girls (56.2%) and 6653 non-Latino white children (52.8%) (Table 2). The median (IQR) age of children with CKD was 6 (1-13) years, and CKD was comparably distributed across age groups (2545 [20.2%] aged <1 year; 2241 [17.8%], 1-3 years; 1515 [12.0%], 4-6 years; 1863 [14.8%], 7-10 years; 1916 [15.2%], 11-14 years; and 2511 [19.9%], 15-17 years). The cohort of participants at risk for CKD included 1 973 258 adults (75.1%). Among them, 955 812 (48.4%) had hypertension alone, while 505 147 (25.6%) had diabetes or prediabetes with hypertension, and 512 299 (26.0%) had diabetes or prediabetes alone. Those at risk for CKD included 1 014 847 women (51.4%), 1 308 036 non-Latino white individuals (66.3%), 60 201 Latino individuals (3.1%), 92 403 black individuals (4.9%), 114 400 Asian individuals (5.8%), 19 820 American Indian and Alaska Native individuals (1.0%), and 11 420 Hawaiian and Pacific Islander individuals (0.6%) (eTable 2 in the Supplement). Proportions of participants at risk for CKD were highest among those aged 50 to 69 years (866 528 [43.9%]).

**Table 1.** Characteristics of Adults With CKD in the CURE-CKD Registry

Characteristic	No. (%)				
	All CKD (N = 606 064)	CKD With Diabetes, Prediabetes, and Hypertension (n = 300 157)	CKD With Hypertension (n = 134 500)	CKD With Diabetes or Prediabetes (n = 81 266)	CKD Alone (n = 90 141)
**Demographic**					
Sex					
Men	267 285 (44.1)	142 197 (47.4)	53 703 (39.9)	38 600 (47.5)	32 785 (36.4)
Women	338 755 (55.9)	157 959 (52.6)	80 795 (60.1)	42 657 (52.5)	57 344 (63.6)
Race/ethnicity					
Non-Latino white	434 474 (71.7)	217 009 (72.3)	106 538 (79.2)	52 453 (64.5)	58 474 (64.9)
Latino	17 625 (2.9)	8749 (2.9)	2570 (1.9)	3339 (4.1)	2967 (3.3)
Black	29 974 (4.9)	17 856 (5.9)	5742 (4.3)	3466 (4.3)	2910 (3.2)
Asian	32 850 (5.4)	19 566 (6.5)	5036 (3.7)	4533 (5.6)	3715 (4.1)
American Indian or Alaska Native	5461 (0.9)	2887 (1.0)	1150 (0.9)	630 (0.8)	794 (0.9)
Hawaiian or Pacific Islander	3899 (0.6)	2509 (0.8)	580 (0.4)	411 (0.5)	399 (0.4)
Other	33 152 (5.5)	15 746 (5.1)	5312 (3.9)	5296 (6.5)	6798 (7.5)
Multiple races	163 (0.1)	120 (0.1)	23 (0.1)	5 (0.1)	15 (0.1)
Not reported^a^	48 466 (8.0)	15 715 (5.2)	7549 (5.6)	11 133 (13.7)	14 069 (15.6)
Entry age, y					
18-39	49 097 (8.1)	11 965 (4.0)	11 389 (8.5)	4797 (5.9)	20 946 (23.2)
40-49	37 544 (6.2)	17 134 (5.7)	7971 (5.9)	4624 (5.7)	7815 (8.7)
50-59	74 616 (12.3)	40 752 (13.6)	14 205 (10.6)	9466 (11.6)	10 193 (11.3)
60-69	129 410 (21.4)	74 535 (24.8)	25 148 (18.7)	16 156 (19.9)	13 571 (15.1)
70-79	144 263 (23.8)	81 043 (27.0)	31 310 (23.3)	18 314 (22.5)	13 596 (15.1)
80-89	127 868 (21.1)	59 896 (20.0)	32 116 (23.9)	20 165 (24.8)	15 691 (17.4)
≥90	43 266 (7.1)	14 832 (4.9)	12 361 (9.2)	7744 (9.5)	8329 (9.2)
**Clinical**					
eGFR CKD category					
1-2	137 784 (22.7)	76 605 (25.5)	25 718 (19.1)	15 882 (19.5)	19 579 (21.7)
3a	226 693 (37.4)	112 931 (37.6)	58 757 (43.7)	27 773 (34.2)	27 232 (30.2)
3b	100 239 (16.5)	48 384 (16.1)	21 642 (16.1)	17 197 (21.2)	13 016 (14.4)
4	39 125 (6.5)	18 737 (6.2)	6083 (4.5)	8862 (10.9)	5443 (6.0)
5, Not dialyzed	20 328 (3.4)	10 181 (3.4)	2861 (2.1)	4652 (5.7)	2634 (2.9)
Not categorized^b^	81 895 (13.5)	33 319 (11.1)	19 439 (14.5)	6900 (8.5)	22 237 (24.7)
UACR, mg/g					
≤30	17 651 (2.9)	12 703 (4.2)	1776 (1.3)	2224 (2.7)	948 (1.1)
>30 to ≤300	27 227 (4.5)	21 435 (7.1)	1089 (0.8)	4066 (5.0)	637 (0.7)
>300	7673 (1.3)	5860 (2.0)	509 (0.4)	995 (1.2)	309 (0.3)
Not measured	553 513 (91.3)	260 159 (86.7)	131 126 (97.5)	73 981 (91.0)	88 247 (97.9)
UPCR, mg/g					
≤150	14 467 (2.4)	7823 (2.6)	2723 (2.0)	2076 (2.6)	1845 (2.0)
>150 to ≤500	5688 (0.9)	3087 (1.0)	1163 (0.9)	763 (0.9)	675 (0.7)
>500	4880 (0.8)	2978 (1.0)	785 (0.6)	696 (0.9)	421 (0.5)
Not measured	581 029 (95.9)	286 269 (95.4)	129 829 (96.5)	77 731 (95.7)	87 200 (96.7)
Age, median (IQR) [No.], y	70 (59-81) [606 064]	70 (60-79) [300 157]	72 (60-83) [134 500]	73 (63-83) [81 266]	64 (42-81) [90 141]
eGFR, median (IQR) [No.], mL/min/1.73 m^2^	53 (41-61) [524 169]	54 (43-63) [266 838]	53 (44-59) [115 061]	49 (35-59) [74 366]	53 (41-66) [67 904]
SBP, mean (SD) [No.], mm Hg	129 (18) [365 561]	131 (18) [202 951]	132 (18) [92 051]	119 (17) [25 533]	119 (16) [45 026]
DBP, mean (SD) [No.], mm Hg	72 (11) [365 561]	72 (10) [202 951]	74 (11) [92 051]	67 (10) [25 533]	70 (10) [45 026]

Abbreviations: CKD, chronic kidney disease; CURE-CKD, Center for Kidney Disease Research, Education, and Hope; DBP, diastolic blood pressure; eGFR, estimated glomerular filtration rate; IQR, interquartile range; SBP, systolic blood pressure; UACR, urine albumin to creatinine ratio; UPCR, urine protein to creatinine ratio.

^a^ Includes null, unknown, and patient did not report.

^b^ Individuals who were identified by CKD administrative codes only could not be categorized.

**Table 2.** Characteristics of Children With CKD in the CURE-CKD Registry

Characteristic	No. (%) (N = 12 591)
**Demographic**	
Sex	
Boys	5511 (43.8)
Girls	7079 (56.2)
Race/ethnicity	
Non-Latino white	6653 (52.8)
Latino	952 (7.6)
Black	677 (5.4)
Asian	697 (5.5)
American Indian or Alaska Native	294 (2.3)
Hawaiian or Pacific Islander	152 (1.2)
Other	2060 (16.4)
Multiple races	12 (0.1)
Not reported^a^	1094 (8.7)
Entry age, y	
<1	2545 (20.2)
1-3	2241 (17.8)
4-6	1515 (12.0)
7-10	1863 (14.8)
11-14	1916 (15.2)
15-17	2511 (19.9)
**Clinical**	
eGFR CKD category	
1-2	2514 (20.0)
3a	1145 (9.1)
3b	503 (4.0)
4	191 (1.5)
5, Not dialyzed	93 (0.7)
Not categorized^b^	8145 (64.7)
UACR, mg/g	
≤30	220 (1.7)
>30 to ≤300	198 (1.6)
>300	102 (0.8)
Not measured	12 071 (95.9)
UPCR, mg/g	
≤150	481 (3.8)
>150 to ≤500	158 (1.3)
>500	159 (1.3)
Not measured	11 793 (93.7)
Age, median (IQR) [No.], y	6 (1-13) [12 591]
eGFR, median (IQR) [No.], mL/min/1.73 m^2^	70 (50-95) [4446]
SBP, mean (SD) [No.], mm Hg	104 (16) [8768]
DBP, mean (SD) [No.], mm Hg	61 (11) [8768]

Abbreviations: CKD, chronic kidney disease; CURE-CKD, Center for Kidney Disease Research, Education, and Hope; DBP, diastolic blood pressure; eGFR, estimated glomerular filtration rate; IQR, interquartile range; SBP, systolic blood pressure; UACR, urine albumin to creatinine ratio; UPCR, urine protein to creatinine ratio.

^a^ Includes null, unknown, and patient did not report.

^b^ Individuals who were identified by CKD administrative codes only could not be categorized.

Comparing adults with CKD with those at risk for CKD, women were more frequently represented in the cohort with CKD than in the cohort at risk for CKD (338 785 [55.9%] vs 1 014 847 [51.4%]; *P* < .001). Non-Latino white individuals (434 474 [71.7%] vs 1 308 036 [66.3%]; *P* < .001) and individuals aged 70 years or older (315 397 [52.0%] vs 386 364 [19.6%]; *P* < .001) were also more common among participants with CKD vs those at risk. There was a higher proportion with rural geolocation within PSJH vs UCLA Health (287 622 [17.2%] vs 6918 [1.8%]; *P* < .001).

### Clinical Characteristics in Adults and Children With CKD and Adults at Risk for CKD

A total of 243 635 adults with CKD (40.2%) were identified by eGFR, 163 375 (27.0%) by administrative codes, and 151 794 (25.0%) by both eGFR and administrative codes. Various combinations of laboratory measurements and administrative codes accounted for the remainder of adult CKD identification. More than half of adults with CKD were in category 3 (3a, 226 693 [37.4%]; 3b, 100 239 [16.5%]) (Table 1). Decreases in prevalence were observed for CKD category 4 (39 125 [6.5%]) and category 5, not dialyzed (20 328 [3.4%]). Median (IQR) eGFR was 53 (41-61) mL/min/1.73 m^2^, and measurements of albuminuria and proteinuria were recorded in 52 511 (8.7%) and 25 035 (4.1%) patients, respectively. Mean (SD) systolic and diastolic blood pressure values were 129 (18) mm Hg and 72 (11) mm Hg, respectively. When participants with CKD and diabetes or prediabetes were assessed separately, higher proportions of patients with diabetes than those with prediabetes had CKD category 4 or 5 (9790 [18.4%] vs 3724 [13.2%]; *P* < .001), and higher levels of albuminuria or proteinuria were present in the group with diabetes compared with the group with prediabetes (5555 [10.4%] vs 965 [3.4%]; *P* < .001) (eTable 3 in the Supplement).

Most children (10 841 [86.1%]) were identified exclusively through CKD administrative codes. Among 8145 children (64.7%), CKD was not categorized (Table 2). Median (IQR) eGFR was 70 (50-95) mL/min/1.73 m^2^. Mean (SD) systolic and diastolic blood pressure were 104 (16) mm Hg and 61 (11) mm Hg, respectively. Measurements of albuminuria and proteinuria were available in 520 (4.1%) and 798 (6.4%) children, respectively.

Median (IQR) eGFR in adults at risk of CKD was 90 (77-103) mL/min/1.73 m^2^, and albuminuria and proteinuria measurements were recorded in 51 470 (2.6%) and 10 285 (0.5%), respectively (eTable 2 in the Supplement). Mean (SD) systolic and diastolic blood pressure values were 135 (18) mm Hg and 79 (12) mm Hg, respectively. When participants with diabetes or prediabetes who were at risk for CKD were analyzed separately, frequency of ascertainment for albuminuria or proteinuria was 7% or less in all groups (eg, among 317 648 patients with diabetes and hypertension, albumin to creatine ratio measurements were available for 21 697 patients [6.8%]; among 187 499 patients with prediabetes and hypertension, protein to creatine ratio measurements were available in 907 [0.5%]) (eTable 4 in the Supplement).

### Prevalence of and Temporal Trends in CKD Among Adults

A total of 12 669 700 patients received care at PSJH (10 793 550 [85.2%]) and UCLA Health (1 876 150 [14.8%]) between January 1, 2006, and December 31, 2017 (eFigure in the Supplement). During this period, 606 064 adults (4.8%) met the CURE-CKD registry entry criteria for CKD. However, when CKD was determined by at least 2 eGFR measurements of less than 60 mL/min/1.73 m^2^ at least 90 days apart, unadjusted prevalence among adults was 26.1% (420 678 of 1 612 737), and adjusted CKD prevalence was 22.6%. When determined by 1 eGFR measure, unadjusted CKD prevalence was 34.4% (873 642 of 2 542 393), and adjusted prevalence was 32.9% (Table 3). Diagnostic coding for CKD was recorded among 171 011 patients (40.7%) with CKD determined by 2 eGFR measurements at least 90 days apart and among 240 630 patients (27.5%) with CKD determined by 1 eGFR measurement.

**Table 3.** Prevalence of CKD Among Adults at PSJH and UCLA Health^a^

Group	2006-2009	2010-2013	2014-2017	2006-2017
Encounters for CURE-CKD criteria, No.				
PSJH and UCLA Health	6 011 129	6 903 084	8 179 860	12 669 700
PSJH	5 366 296	6 100 530	7 025 762	10 793 550
UCLA Health	644 833	802 554	1 154 098	1 876 150
2 eGFR measures, No.^b^				
PSJH and UCLA Health	288 258	913 177	1 076 536	1 612 737
PSJH	199 789	809 519	895 259	1 356 375
UCLA Health	88 469	103 658	181 277	256 480
1 eGFR measure, No.				
PSJH and UCLA Health	577 845	1 500 025	1 893 309	2 542 393
PSJH	430 252	1 320 786	1 582 785	2 115 250
UCLA Health	147 593	179 239	310 524	427 143
CKD by CURE-CKD criteria, No. (%)				
PSJH and UCLA Health	93 644 (1.6)	393 455 (5.7)	683 574 (8.4)	606 064 (4.8)
PSJH	69 332 (1.3)	337 748 (5.5)	576 503 (8.2)	505 278 (4.7)
UCLA Health	24 312 (3.8)	55 707 (6.9)	107 071 (9.3)	100 786 (5.4)
CKD by 2 eGFR measures^b^				
PSJH and UCLA Health, No. (%)	87 225 (30.3)	258 130 (28.3)	238 750 (22.2)	420 678 (26.1)
Adjusted, %	20.8	22.6	21.2	22.6
Diagnosis code, No. (%)	2766 (3.2)	65 213 (25.3)	124 897 (52.3)	171 011 (40.7)
PSJH, No. (%)	64 090 (32.1)	233 369 (28.8)	205 427 (22.9)	363 365 (26.8)
Adjusted %	21.2	23.2	21.7	23.2
Diagnosis code, No. (%)	2287 (3.6)	56 631 (23.9)	105 447 (51.3)	144 956 (39.9)
UCLA Health, No. (%)	23 135 (26.1)	24 734 (13.7)	33 323 (18.3)	57 313 (22.3)
Adjusted %	19.2	19.6	18.8	20.0
Diagnosis code, No. (%)	479 (2.1)	8582 (34.7)	19 450 (58.4)	26 055 (45.5)
CKD by 1 eGFR measure				
PSJH and UCLA Health, No. (%)	170 813 (29.6)	473 685 (31.6)	526 793 (27.8)	873 642 (34.4)
Adjusted %	22.3	27.8	28.5	32.9
Diagnosis code, No. (%)	3304 (1.9)	81 309 (17.2)	193 927 (36.8)	240 630 (27.5)
PSJH, No. (%)	127 351 (29.6)	427 642 (32.4)	458 334 (29.0)	751 690 (35.5)
Adjusted %	21.8	28.8	29.4	34.0
Diagnosis code, No. (%)	2732 (2.1)	69 985 (16.4)	161 180 (35.2)	200 061 (26.6)
UCLA Health, No. (%)	43 362 (29.4)	46 043 (25.7)	68 459 (22.0)	121 952 (28.6)
Adjusted %	23.3	22.6	24.2	27.5
Diagnosis code, No. (%)	572 (1.3)	11 324 (24.6)	32 747 (47.8)	40 569 (33.2)

Abbreviations: CKD, chronic kidney disease; CURE-CKD, Center for Kidney Disease Research, Education, and Hope; eGFR, estimated glomerular filtration rate; PSJH, Providence St Joseph Health; UCLA, University of California, Los Angeles.

^a^ *P* < .001 for all unadjusted and adjusted comparisons.

^b^ Calculated by CKD-Epidemiology equation from the mean of at least 2 serum creatinine measurements at least 90 days apart.

**Figure 1.**
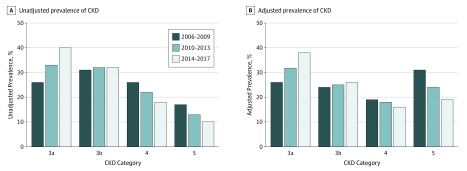
Prevalence of Chronic Kidney Disease (CKD) Category 3a, 3b, 4, and 5 in 2006 to 2009, 2010 to 2013, and 2014 to 2017 A, Unadjusted prevalence. B, Prevalence adjusted by age, sex, and race/ethnicity.

Temporal trends in CKD prevalence were determined for the 3 following periods: 2006 to 2009, 2010 to 2013, and 2014 to 2017. CKD prevalence rates by CURE-CKD registry criteria increased over time (2006-2009, 93 644 of 6 011 129 [1.6%]; 2010-2013, 393 455 of 6 903 084 [5.7%]; and 2014-2017, 683 574 of 8 179 860 [8.4%]). Prevalence rates adjusted for age, sex, and race/ethnicity and based on eGFR classification alone were higher and stable over time among patients with 2 or more eGFR measurements at least 90 days apart (20.8%, 22.6%, and 21.2%, respectively), while increasing adjusted prevalence was observed among patients with 1 eGFR measurement (22.3%, 27.8%, and 28.5%, respectively). Rates of administrative coding for CKD increased progressively at both PSJH and UCLA Health (Table 3). For example, among patients with 2 eGFR measurements of less than 60 mL/min/1.73 m^2^ at least 90 days apart, 2766 of 87 225 (3.2%) were identified by administrative code during 2006 to 2009 and 124 897 of 238 750 (52.3%) were identified by administrative code during 2014 to 2017. When CKD categories were analyzed by at least 2 eGFR measurements at least 90 days apart, unadjusted prevalence rates and prevalence rates adjusted by age, sex, and race/ethnicity showed progressive increases for categories 3a and 3b with declines in categories 4 and 5 (eg, category 3a: 2006-2009, 22 805 [prevalence, 26.1%; adjusted prevalence 26.1%]; 2014-2017, 96 449 [prevalence 40.4%; adjusted prevalence, 38.2%]; category 4: 2006-2009, 22 338 [prevalence, 25.7%, adjusted prevalence, 19.4%], 2014-2017, 42 065 [prevalence, 17.6%; adjusted prevalence, 16.1%]) (Figure 1).

### Prescription Medication Use and Temporal Trends in Adults With CKD

Angiotensin-converting enzyme (ACE) inhibitors or angiotensin receptor blockers (ARBs) were prescribed to 127 574 adults (20.5%) with CKD, with slightly higher use of these agents among those with CKD and hypertension (112 449 of 434 657 [25.9%]) (eTable 5 in the Supplement). By contrast, 204 307 participants (33.7%) with CKD had prescriptions for nonsteroidal anti-inflammatory drugs (NSAIDs) or proton pump inhibitors (PPIs). Statins and aspirin were prescribed to 107 445 (17.7%) and 110 335 (18.2%) individuals, respectively. The most commonly prescribed antihyperglycemic agents among patients with CKD and diabetes or prediabetes were insulin (38 278 [10.0%]), metformin (30 393 [7.9%]), and sulfonylureas (16 989 [4.4%]). Medications prescribed among the cohort of participants at risk of CKD were generally similar to the CKD cohort, except for more common use of insulin (83 363 [16.3%]) among those with diabetes and of NSAIDs (701 493 [35.5%]) and PPIs (295 804 (15.0%]) overall (eTable 6 in the Supplement).

**Figure 2.**
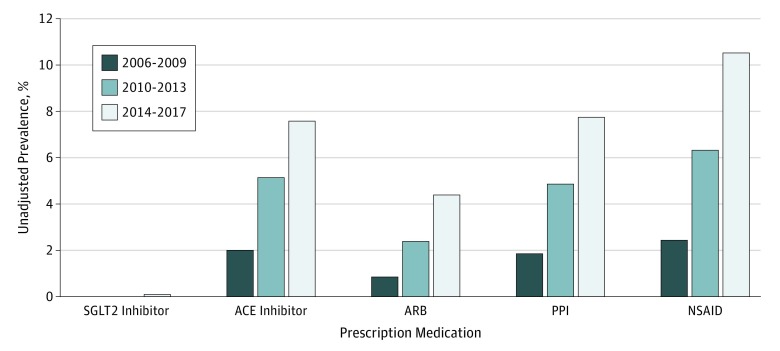
Prevalence of Prescription Medication Use in Chronic Kidney Disease Categories 3a to 5 in 2006 to 2009, 2010 to 2013, 2014 to 2017 ACE indicates angiotensin-converting enzyme; ARB, angiotensin receptor blocker; NSAID, nonsteroidal anti-inflammatory drug; PPI, proton pump inhibitor; and SGLT2, sodium-glucose cotransporter 2.

Temporal trends in prescription medications were determined for participants with CKD determined by 2 eGFR measurements of less than 60 mL/min/1.73 m^2^ at least 90 days apart for the 3 periods. Use rates of ACE inhibitors, ARBs, NSAIDs, and PPIs across CKD categories 3a to 5 all increased (ACE inhibitors: 2006-2009, 5654 [2.0%]; 2010-2013, 46 921 [5.1%]; 2014-2017, 81 601 [7.6%]; ARBs: 2006-2009, 2461 [0.9%]; 2010-2013, 21 791 [2.4%]; 2014-2017, 47 233 [4.4%]; NSAIDs: 2006-2009, 7009 [2.4%]; 2010-2013, 57 705 [6.3%]; 2014-2017, 113 251 [11.0%]; PPIs: 2006-2009, 5331 [1.8%]; 2010-2013, 44 362 [4.9%]; 2014-2017, 83 340 [7.7%]) (Figure 2). Sodium-glucose cotransporter 2 inhibitors were rarely prescribed, but use increased over time (2006-2009, 0; 2010-2013, 22 [0.002%]; 2014-2017, 1002 [0.093%]).

## Discussion

More than 2.6 million adults and children who received care at PSJH and UCLA Health from 2006 to 2017 had CKD or were at risk of CKD. Overall, CKD prevalence among adults in the health care systems was 4.8%, as determined by a combination of eGFR, albuminuria and proteinuria measures, and administrative code criteria. However, adult CKD prevalence adjusted for age, sex, and race/ethnicity was 22.6% based on persistently low eGFR alone. Adults with CKD were more likely to be older, women, and non-Latino white individuals. In this study, CKD category 3 was most frequent, with a clear drop-off in prevalence at more advanced categories. Kidney protective agents (ie, renin-angiotensin system inhibitors) were prescribed to approximately one-fifth of adults with CKD, while potential nephrotoxins (ie, NSAIDs and PPIs) were prescribed to more than one-third of adults with CKD. Albuminuria and proteinuria testing for CKD assessment was rarely reported.

The CURE-CKD registry is among the most comprehensive CKD registries worldwide. A unique feature is the extensive amount of patient-level data on laboratory measures, prescriptions, and vital signs, combined with administrative codes, to identify CKD and major risk factors according to guideline-based criteria.^21,24,25^ Previous registries were restricted by containing primarily administrative data, ESKD, primary care practices, single health care systems, older adults, or men.^4,26,27,28,29,30,31,32^ In contrast, CURE-CKD participants represent the life span, from children to adults, and include women and men and a wide spectrum of races and ethnicities across an expansive region of the western United States that has not been previously involved in large-scale epidemiologic studies of CKD. Moreover, PSJH and UCLA Health care for patients in a variety of settings that include academic, primary care, and specialty practices as well as community health and safety-net systems. Rural patients were well represented in the geography covered by PSJH. Thus, CURE-CKD provides in-depth identification of patients with and at risk for CKD in contemporary US health care systems.

In CURE-CKD, the progressive increase in adult CKD prevalence was largely driven by diagnostic coding. Among adults with persistently low eGFR, use of CKD administrative codes increased from 3.2% to 52.3% between the periods of 2006 to 2009 and 2014 to 2017, while overall CKD prevalence estimates, adjusted for age, sex, and race/ethnicity, were essentially stable between 20.8% and 22.6%. Although the upward trend in CKD recognition represents a clinically meaningful improvement, nearly one-half of patients with low eGFR remained undiagnosed in the most recent period. The present findings from CURE-CKD point to the critical need for quality improvement and research at the point of care.

Although nearly two-thirds of the adults with CKD had diabetes, hypertension, or prediabetes, rates of laboratory testing for albuminuria or proteinuria and of prescribing ACE inhibitors or ARBs were low. Potentially nephrotoxic agents (ie, NSAIDs and PPIs) were used more commonly than renin-angiotensin system inhibitors. Given the most common cause of death in CKD is cardiovascular disease, the low use of preventive agents, such as statins and aspirin, is also concerning.^33,34^ Compared with participants in the National Health and Nutrition Examination Survey, patients with CKD in CURE-CKD received ACE inhibitors or ARBs much less often during approximately the same period.^35^ Although CURE-CKD found an increase in uptake of renin-angiotensin system inhibitors in adults with CKD categories 3a to 5, NSAID and PPI use also increased over time. However, these prescription rates were lower than in the overall CKD cohort, perhaps because of concerns about adverse effects with more advanced CKD. While this may seem counterintuitive for renin-angiotensin system inhibitors, these agents may be avoided because of fear of complications such as hyperkalemia or acute kidney injury, especially in acute care settings. In Ontario, Canada, primary care practices reported ACE inhibitor or ARB use in three-fourths of patients with CKD, but the metric was confined to those with diabetes and albuminuria or adults older than 66 years.^30,31^ Nevertheless, rates of albuminuria testing in the overall CKD population were comparably low, although avoidance of NSAIDs was better among patients in Canada than in CURE-CKD.^30^ A recent Canada-wide study^32^ from an EHR-based surveillance system in primary care found that only 4 of 12 quality indicators for CKD care were met, with ACE inhibitor or ARB use among approximately one-third of patients with diabetes or proteinuria. Contrasts exist between reports from health care systems, community screenings, primary care practices, and countries, but they consistently illuminate major gaps in CKD care and the need for more comprehensive surveillance to uncover actionable trends.

In comparison with patients treated at PSJH and UCLA Health, the Kidney Early Evaluation Program and the National Health and Nutrition Examination Survey have reported lower frequencies of individuals at risk for CKD in community screenings.^36^ Moreover, associations between risk factors and CKD are remarkably complex. For example, although a primary contributor to CKD is diabetes, CKD in patients with diabetes greatly amplifies cardiovascular risks.^37^ Additionally, nearly one-fifth of patients with CKD in the CURE-CKD registry had prediabetes. The prediabetes phenotype of CKD appears less severe than the diabetes phenotype of CKD, as reflected by fewer patients with advanced CKD categories, albuminuria, or proteinuria and prediabetes. Nevertheless, consistent with findings from the National Health and Nutrition Examination Survey, findings from CURE-CKD support the observation that subdiabetic hyperglycemia may contribute to kidney damage before overt diabetes ensues.^13^ The CURE-CKD registry contains abundant longitudinal data that will be invaluable for elucidating CKD incidence among individuals at risk as well as progression and complications in those with CKD. Given its vast scope, CURE-CKD is also ideally suited to generate and validate CKD risk prediction models.^38^

### Strengths and Limitations

Strengths of the CURE-CKD registry include the large sample size, long observation duration, and curated patient-level data from 2 US health care systems. However, this study has limitations. First, CURE-CKD is limited by differences in documentation methods across and between health care systems and varying attrition rates based on insurance, socioeconomic factors, and geolocation. Variation in platforms even within a common EHR system also presents a limitation to the creation of interinstitutional registries, highlighting the importance of collaboration in identifying data elements, structures, and synchronization. Lack of information on over-the-counter medications underestimates the usage rates for NSAIDs, PPIs, and other potential nephrotoxins. Data in CURE-CKD on sodium-glucose cotransporter 2 inhibitor use, recently recommended for diabetes and CKD, came from an era before this new indication. It will be important to follow this trend to ensure sodium-glucose cotransporter 2 inhibitors are delivered to patients who may benefit. Another limitation of EHR-based registries is undercoding and miscoding. To mitigate this limitation, patient-level data for laboratory values, vital signs, and prescriptions were used to classify CURE-CKD registry participants and their care, which allowed for the use of guideline-based criteria for persistence of low eGFR or elevated albuminuria or proteinuria levels. Although CURE-CKD produced a lower range estimate of overall CKD prevalence compared with other US reports, this prevalence rate is similar to that found in Canadian primary care.^4,32^ A higher range estimate for CKD based solely on eGFR could be because of more frequent testing in patients with higher risk who were treated by both specialty and primary care practices at PSJH and UCLA Health. Ascertainment bias is an inherent limitation of EHR-based registries, and information about CKD will also be missed from patients receiving care elsewhere or not receiving testing. The actual prevalence of overall CKD likely lies between the low (4.8%) and high (22.6%) range estimations from CURE-CKD. Nevertheless, these detailed prevalence estimates are strengths that represent the complexity and composition of patients treated in typical US health care systems.

## Conclusions

In conclusion, the CURE-CKD registry reveals a burgeoning number of patients with CKD and major risk factors of diabetes, hypertension, and prediabetes. Rates of identification and use of kidney protective agents were low, while nephrotoxin use was widespread, underscoring the pressing need for practice-based improvement in CKD prevention, recognition, and treatment. These real-world data lay the groundwork for the development of more effective strategies to deliver care that enhances wellness and survival for patients with and at risk for CKD.

## Notes

Supplement. **eFigure. **CURE-CKD STROBE Diagram, Participant Groups by CKD and At-Risk Categories **eTable 1. **Inclusion Criteria for the CURE-CKD Registry **eTable 2. **Characteristics of Adults at Risk of CKD in the CURE-CKD Registry **eTable 3. **Characteristics of Adults With CKD and Diabetes or Prediabetes With or Without Hypertension in the CURE-CKD Registry **eTable 4. **Characteristics of Adults at Risk of CKD With Diabetes or Prediabetes With or Without Hypertension in the CURE-CKD Registry **eTable 5. **Medications Prescribed to Adults With CKD in the CURE-CKD Registry **eTable 6. **Medications Prescribed to Adults at Risk of CKD in the CURE-CKD Registry
