# Supplementary material for: Clinical Characteristics of and Risk Factors for Chronic Kidney Disease Among Adults and Children: An Analysis of the CURE-CKD Registry
Source: JAMA Netw Open. 2019 Dec 20;2(12):e1918169. doi: 10.1001/jamanetworkopen.2019.18169 (PMC6991307; doi:10.1001/jamanetworkopen.2019.18169)
Supplement: Supplement. — eFigure. CURE-CKD STROBE Diagram, Participant Groups by CKD and At-Risk Categories eTable 1. Inclusion Criteria for the CURE-CKD Registry eTable 2. Characteristics of Adults at Risk of CKD in the CURE-CKD Registry eTable 3. Characteristics of Adults With CKD and Diabetes or Prediabetes With or Without Hypertension in the CURE-CKD Registry eTable 4. Characteristics of Adults at Risk of CKD With Diabetes or Prediabetes With or Without Hypertension in the CURE-CKD Registry eTable 5. Medications Prescribed to Adults With CKD in the CURE-CKD Registry eTable 6. Medications Prescribed to Adults at Risk of CKD in the CURE-CKD Registry [file jamanetwopen-2-e1918169-s001.pdf]

Supplementary Online Content

Tuttle KR, Alicic RZ, Duru OK, et al. Clinical characteristics of and risk factors for chronic kidney disease among adults and children: an analysis of the CURE-CKD registry. *JAMA Netw Open*. 2019;2(12):e1918169. doi:10.1001/jamanetworkopen.2019.18169

eFigure. CURE-CKD STROBE Diagram, Participant Groups by CKD and At-Risk Categories
eTable 1. Inclusion Criteria for the CURE-CKD Registry
eTable 2. Characteristics of Adults at Risk of CKD in the CURE-CKD Registry
eTable 3. Characteristics of Adults With CKD and Diabetes or Prediabetes With or Without Hypertension in the CURE-CKD Registry
eTable 4. Characteristics of Adults at Risk of CKD With Diabetes or Prediabetes With or Without Hypertension in the CURE-CKD Registry
eTable 5. Medications Prescribed to Adults With CKD in the CURE-CKD Registry
eTable 6. Medications Prescribed to Adults at Risk of CKD in the CURE-CKD Registry

This supplementary material has been provided by the authors to give readers additional information about their work.

© 2019 Tuttle KR et al. *JAMA Network Open.*

eFigure. CURE-CKD STROBE Diagram, Participant Groups by CKD and At-Risk Categories

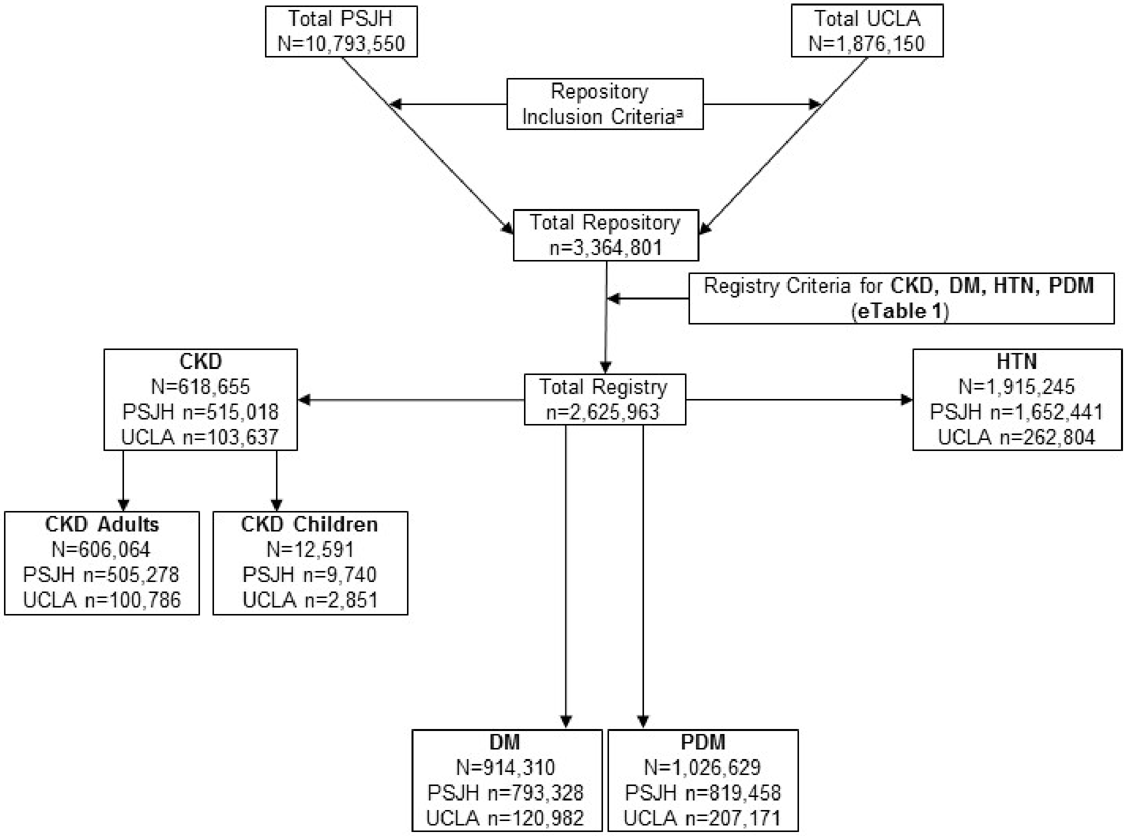

[a]The repository includes any measurement of estimated glomerular filtration rate <60 mL/min/1.73m^2, urine-to-creatinine ratio ≥30 mg/g, urine total protein-to-creatinine ratio ≥150 mg/g, systolic blood pressure ≥140 mm Hg, diastolic blood pressure ≥90 mm Hg, hemoglobin A1c ≥5·7%, random blood glucose ≥140 mg/dL, fasting blood glucose ≥100 mg/dL, prescription for an anti-hyperglycemic agent, or an encounter with a CKD, DM, HTN, or PDM administrative code.

© 2019 Tuttle KR et al. *JAMA Network Open.*

eTable 1. Inclusion Criteria for the CURE-CKD Registry

Disease Type	Inclusion Criteria
**Chronic Kidney Disease**[21]	At least two eGFR (CKD-EPI equation – adults; beside Schwartz equation – children) measurements <60 mL/min/1.73m^2 at least 90 days apart, OR<br>At least two laboratory measurements at least 90 days apart where albuminuria or proteinuria was indicated by UACR >30 mg/g or UPCR >150 mg/g, OR<br>At least one eGFR and one albuminuria or proteinuria measurement meeting the above requirements at least 90 days apart, OR<br>At least one encounter (inpatient or outpatient) with an ICD-9 or ICD-10 diagnosis code indicating chronic kidney disease is present
**Hypertension**[25]	At least two vital sign measurements >14 days apart with a systolic blood pressure ≥140 mm Hg or diastolic blood pressure ≥90 mm Hg, OR<br>At least one encounter (inpatient or outpatient) with an ICD-9 or ICD-10 diagnosis code indicating hypertension is present
**Diabetes Mellitus**[23]	Any qualifying laboratory result:<br>1 HbA1c ≥6.5%, OR<br>2 random blood glucose measurements ≥200 mg/dL, at least one day, but no more than two years apart, OR<br>2 fasting blood glucose measurements ≥126 mg/dL, at least one day, but no more than two years apart, OR<br>At least one medication record with a pharmaceutical class (i.e. anti-hyperglycemic medications) for treating diabetes mellitus (excludes individuals diagnosed with polycystic ovarian syndrome taking metformin), OR<br>At least two outpatient ICD-9 or ICD-10 diagnosis codes indicating diabetes mellitus is present, OR<br>At least one inpatient ICD-9 or ICD-10 diagnosis code indicating diabetes mellitus is present
**Pre-Diabetes Mellitus**[23]	Any qualifying laboratory result:<br>1 HbA1c 5.7–6·4%, OR<br>2 random blood glucose measurements 140–199 mg/dL, at least one day, but no more than two years apart, OR<br>2 fasting blood glucose measurements 100–125 mg/dL, at least one day, but no more than two years apart, OR<br>At least one encounter (inpatient or outpatient) with an ICD-9 or ICD-10 diagnosis code indicating pre-diabetes mellitus

© 2019 Tuttle KR et al. *JAMA Network Open.*

eTable 2. Characteristics of Adults at Risk of CKD in the CURE-CKD Registry

	All At-Risk N=1,973,258	DM-PDM/HTN N=505,147	HTN N=955,812	DM-PDM N=512,299
**Demographics**	n (%)			
**Gender**				
Men	957,921 (49)	247,748 (49)	472,750 (49)	237,423 (46)
Women	1,014,847 (51)	257,388 (51)	482,700 (51)	274,759 (54)
**Race**				
White Non-Latino	1,308,036 (66)	347,232 (69)	672,628 (70)	288,176 (56)
White Latino	60,201 (3)	15,062 (3)	24,244 (3)	20,796 (4)
Black	92,403 (5)	26,962 (5)	44,645 (5)	20,801 (4)
Asian	114,400 (6)	33,983 (7)	41,656 (4)	38,761 (8)
American Indian/Alaskan	19,820 (1)	5,413 (1)	9,669 (1)	4,738 (1)
Hawaiian/Pacific Islander	11,420 (1)	3,566 (1)	5,011 (1)	2,843 (1)
Other	159,514 (8)	36,200 (7)	70,422 (7)	52,892 (10)
Multiple Races	511 (<1)	128 (<1)	255 (<1)	128 (<1)
Not Reported[a]	206,953 (10)	36,601 (7)	87,282 (9)	83,070 (16)
**Entry Age (years)**				
18-39	403,301 (20)	62,905 (12)	242,008 (25)	98,388 (19)
40-49	317,065 (16)	75,309 (15)	161,096 (17)	80,660 (16)
50-59	442,131 (22)	127,502 (25)	201,768 (21)	112,861 (22)
60-69	424,397 (22)	133,992 (27)	180,893 (19)	109,512 (21)
70-79	244,670 (12)	73,348 (15)	106,100 (11)	65,222 (13)
80-89	114,967 (6)	27,457 (5)	51,276 (5)	36,234 (7)
90+	26,727 (1)	4,634 (1)	12,671 (1)	9,422 (2)
**Clinical characteristics**				
**UACR**				
≤ 30 mg/g	43,348 (2)	21,709 (4)	3,876 (<1)	17,763 (3)
> 30 and ≤ 300 mg/g	7,035 (<1)	3,238 (1)	359 (<1)	3,438 (1)
> 300 mg/g	1,095 (<1)	434 (<1)	39 (<1)	622 (<1)
Not Measured	1,921,780 (97)	479,766 (95)	951,538 (100)	490,476 (96)
**UPCR**				
≤ 150 mg/g	5,851 (<1)	1,585 (<1)	1,792 (<1)	2,474 (<1)
> 150 and ≤ 500 mg/g	3,676 (<1)	1,090 (<1)	1,343 (<1)	1,243 (<1)
> 500 mg/g	758 (<1)	208 (<1)	231 (<1)	319 (<1)
Not Measured	1,962,973 (99)	502,264 (99)	952,446 (100)	508,263 (99)
	median, IQR (n)			
**Age (years)**	56, 43-67 (1,973,258)	59, 48-68 (505,147)	53, 39-65 (955,812)	57, 44-68 (512,299)
**eGFR (mL/min/1.73m²)**	90, 77-103 (974,776)	90, 78-101 (313,597)	90, 77-103 (333,737)	90, 74-104 (327,442)
	mean±SD (n)			
**SBP (mm Hg)**	135±18 (1,547,665)	134±16 (392,609)	139±17 (912,488)	123±16 (242,568)
**DBP (mm Hg)**	79±12 (1,547,665)	78±11 (392,609)	82±11 (912,488)	72±10 (242,568)

CKD-chronic kidney disease; CURE-CKD-Center for Kidney Disease Research, Education, and Hope; DM-diabetes mellitus; PDM-pre-diabetes mellitus; HTN-hypertension; N-overall sample size; n-by group sample size; UACR-urine albumin-to-creatinine ratio; UPCR-urine protein-to-creatinine ratio; SD-standard deviation; eGFR-estimated glomerular filtration rate; SBP-systolic blood pressure; DBP-diastolic blood pressure; [a]includes null, unknown, and patient did not report

© 2019 Tuttle KR et al. *JAMA Network Open.*

eTable 3. Characteristics of Adults With CKD and Diabetes or Prediabetes With or Without Hypertension in the CURE-CKD Registry

	CKD/DM/HTN N=226,367	CKD/DM N=53,186	CKD/PDM/HTN N=73,790	CKD/PDM N=28,080
**Clinical characteristics**	**n (%)**			
**eGFR Category**				
CKD 1-2	59,963 (26)	10,704 (20)	16,646 (23)	5,178 (18)
CKD 3a	77,911 (34)	16,357 (31)	35,017 (47)	11,416 (41)
CKD 3b	36,820 (16)	11,263 (21)	11,560 (16)	5,934 (21)
CKD 4	15,323 (7)	6,252 (12)	3,416 (5)	2,610 (9)
CKD 5 not dialyzed	8,925 (4)	3,538 (7)	1,255 (2)	1,114 (4)
Not Categorized[a]	27,425 (12)	5,072 (9)	5,894 (8)	1,828 (7)
**UACR**				
≤ 30 mg/g	11,133 (5)	1,746 (3)	1,570 (2)	478 (2)
> 30 and ≤ 300 mg/g	20,276 (9)	3,644 (7)	1,159 (2)	422 (2)
> 300 mg/g	5,487 (2)	853 (2)	373 (1)	142 (1)
Not Measured	189,471 (84)	46,943 (88)	70,688 (96)	27,038 (96)
**UPCR**				
≤ 150 mg/g	6,188 (3)	1,450 (3)	1,635 (2)	626 (2)
> 150 and ≤ 500 mg/g	2,490 (1)	547 (2)	597 (1)	216 (1)
> 500 mg/g	2,437 (1)	511 (<1)	541 (<1)	185 (<1)
Not Measured	215,252 (95)	50,678 (95)	71,017 (96)	27,053 (96)
	**median, IQR (n)**			
**Age (years)**	69, 60-78 (226,367)	71, 60-82 (53,186)	73, 63-83 (73,790)	76, 63-86 (28,080)
**eGFR (mL/min/1.73m2)**	54, 42-64 (198,942)	48, 34-59 (48,114)	54, 46-60 (67,896)	51, 38-59 (26,252)
**HbA1c (%)**	6.8, 6.1-7.9 (84,571)	6.8, 6.2-7.9 (19,207)	5.8, 5.6-6 (12,428)	5.8, 5.6-6 (5,536)
	**mean±SD (n)**			
**SBP (mm Hg)**	131±18 (155,840)	120±18 (18,271)	131±17 (47,111)	117±15 (7,262)
**DBP (mm Hg)**	71±11 (155,840)	67±10 (18,271)	73±10 (47,111)	68±9 (7,262)

CKD-chronic kidney disease; DM-diabetes mellitus; PDM-pre-diabetes mellitus; HTN-hypertension; CURE-CKD-Center for Kidney Disease Research, Education, and Hope; N-overall sample size; n-by group sample size; eGFR-estimated glomerular filtration rate; UACR-urine albumin-to-creatinine ratio; UPCR-urine protein-to-creatinine ratio; IQR-interquartile range; SD-standard deviation; SBP-systolic blood pressure; DBP-diastolic blood pressure; Hb-hemoglobin; [a]CKD Not Categorized-CKD administrative code only

© 2019 Tuttle KR et al. *JAMA Network Open.*

eTable 4. Characteristics of Adults at Risk of CKD With Diabetes or Prediabetes With or Without Hypertension in the CURE-CKD Registry

	DM/HTN N=317,648	DM N=304,416	PDM/HTN N=187,499	PDM N=207,883
**Clinical characteristics**	n (%)			
**UACR**				
≤ 30 mg/g	18,352 (6)	13,722 (5)	3,357 (2)	4,041 (2)
> 30 and ≤ 300 mg/g	2,932 (1)	2,907 (1)	306 (<1)	531 (<1)
> 300 mg/g	413 (<1)	549 (<1)	21 (<1)	73 (<1)
Not Measured	295,951 (93)	287,238 (94)	183,815 (98)	203,238 (98)
**UPCR**				
≤ 150 mg/g	1,105 (<1)	1,708 (1)	480 (<1)	766 (<1)
> 150 and ≤ 500 mg/g	712 (<1)	759 (<1)	378 (<1)	484 (<1)
> 500 mg/g	159 (<1)	250 (<1)	49 (<1)	69 (<1)
Not Measured	315,672 (99)	301,699 (99)	186,592 (100)	206,564 (99)
	median, IQR (n)			
**Age (years)**	59, 49-68 (317,648)	58, 45-69 (304,416)	58, 47-67 (187,499)	54, 41-65 (207,883)
**eGFR (mL/min/1.73m2)**	90, 77-101 (194,562)	89,71-103 (170,070)	89, 78-100 (119,035)	91, 77-104 (157,372)
**HbA1c (%)**	6.6, 6.0-7.8 (101,804)	7.0, 6.4-8.5 (92,040)	5.8, 5.7-6.0 (59,662)	5.8, 5.7-6.0 (126,542)
	mean±SD (n)			
**SBP (mm Hg)**	133±17 (244,034)	125±17 (172,172)	135±16 (148,575)	118±13 (70,396)
**DBP (mm Hg)**	76±11 (244,034)	72±10 (172,172)	80±11 (148,575)	71±9 (70,396)

CKD-chronic kidney disease; DM-diabetes mellitus; PDM-pre-diabetes mellitus; HTN-hypertension; CURE-CKD-Center for Kidney Disease Research, Education, and Hope; N-overall sample size; n-by group sample size; UACR-urine albumin-to-creatinine ratio; UPCR-urine protein-to-creatinine ratio; SD-standard deviation; eGFR-estimated glomerular filtrations rate; SBP-systolic blood pressure; DBP-diastolic blood pressure; IQR-interquartile range; Hb-hemoglobin

© 2019 Tuttle KR et al. *JAMA Network Open.*

eTable 5. Medications Prescribed to Adults With CKD in the CURE-CKD Registry

Entry Medications	All CKD N=606,064	CKD/DM-PDM/HTN N=300,157	CKD/HTN N=134,500	CKD/DM-PDM N=81,266	CKD Only N=90,141
	n (%)				
**Anti-hypertensive**					
ACE inhibitor	84,687 (14)	51,286 (17)	25,111 (19)	4,118 (5)	4,172 (5)
ARB	39,888 (7)	24,218 (8)	11,834 (9)	1,825 (2)	2,011 (2)
DRI	13,566 (2)	8,176 (3)	4,395 (3)	464 (1)	531 (1)
MRA	3,157 (1)	1,702 (1)	814 (1)	287 (<1)	354 (<1)
Diuretic	97,457 (16)	56,235 (19)	28,830 (21)	5,813 (7)	6,579 (7)
Beta-blocker	88,705 (15)	50,636 (17)	27,942 (21)	4,427 (5)	5,700 (6)
CCB	57,635 (10)	33,989 (11)	18,074 (13)	2,495 (3)	3,077 (3)
Alpha blocker	49,797 (8)	27,421 (9)	14,868 (11)	3,264 (4)	4,244 (5)
Central sympatholytic	13,951 (2)	8,021 (3)	4,169 (3)	767 (1)	994 (1)
Other vasodilator[a]	14,554 (2)	8,656 (3)	3,929 (3)	981 (1)	988 (1)
**Anti-hyperglycemic**					
Insulin	38,278 (6)	31,820 (11)	0 (0)	6,458 (8)	0 (0)
Metformin	30,393 (5)	26,778 (9)	28 (<1)	3,564 (4)	23 (<1)
Sulfonylurea	16,989 (3)	14,912 (5)	0 (0)	2,077 (3)	0 (0)
TZD	1,182 (<1)	1,108 (<1)	0 (0)	74 (<1)	0 (0)
DPP-4 inhibitor	3,560 (1)	3,075 (1)	0 (0)	485 (1)	0 (0)
GLP-1 receptor agonist	1,430 (<1)	1,247 (<1)	0 (0)	183 (<1)	0 (0)
SGLT-2 inhibitor	406 (<1)	323 (<1)	0 (0)	83 (<1)	0 (0)
**Cardiovascular risk reduction**					
Statin	107,445 (18)	64,542 (22)	28,897 (21)	6,942 (9)	7,064 (8)
ASA	110,335 (18)	60,855 (20)	34,085 (25)	6,424 (8)	8,971 (10)
Other anti-platelet agents	18,052 (3)	10,883 (4)	4,922 (4)	1,144 (1)	1,104 (1)
**Nephrotoxins**					
NSAID	124,442 (21)	54,732 (18)	40,128 (30)	7,111 (9)	22,471 (25)
PPI	79,865 (13)	41,993 (14)	23,991 (18)	5,772 (7)	8,109 (9)

CKD-chronic kidney disease; CURE-CKD-Center for Kidney Research, Education, and Hope; DM-diabetes mellitus; PDM-pre-diabetes mellitus; HTN-hypertension; N-overall sample size; n-prescription by group (non-mutually exclusive, i.e. participants may be on multiple/combination medications); ACE-angiotensin converting enzyme; ARB-angiotensin II receptor blocker; DRI-direct renin inhibitor; MRA-mineralocorticoid receptor antagonist; CCB-calcium channel blocker; TZD-thiazolidinedione; DPP-dipeptidyl peptidase; GLP-glucagon-like peptide; SGLT-sodium glucose co-transporter; ASA-acetylsalicylic acid; NSAID-nonsteroidal anti-inflammatory drug; PPI-proton pump inhibitor; [a]Other vasodilators-hydralazine and minoxidil

© 2019 Tuttle KR et al. *JAMA Network Open.*

eTable 6. Medications Prescribed to Adults at Risk of CKD in the CURE-CKD Registry

Baseline Medications	All At-Risk N=1,973,258	DM-PDM/HTN N=505,147	HTN N=955,812	DM-PDM N=512,299
	n (%)			
**Anti-hypertensive**				
ACE inhibitor	298,353 (15)	102,042 (20)	144,218 (15)	52,093 (10)
ARB	127,814 (6)	43,404 (9)	62,190 (7)	22,220 (4)
DRI	56,183 (3)	19,322 (4)	30,194 (3)	6,667 (1)
MRA	6,367 (<1)	1,840 (<1)	2,735 (<1)	1,792 (<1)
Diuretic	260,524 (13)	87,340 (17)	128,655 (13)	44,529 (9)
Beta-blocker	311,387 (16)	99,275 (20)	153,510 (16)	58,602 (11)
CCB	157,735 (8)	52,415 (10)	80,739 (8)	24,581 (5)
Alpha blocker	184,675 (9)	52,721 (10)	91,739 (10)	40,216 (8)
Central sympatholytic	39,793 (2)	11,465 (2)	22,303 (2)	6,025 (1)
Other vasodilator[a]	61,440 (3)	20,023 (4)	21,790 (2)	19,627 (4)
**Anti-hyperglycemic**				
Insulin	145,284 (7)	61,921 (12)	0 (0)	83,363 (16)
Metformin	161,631 (8)	71,713 (14)	706 (<1)	89,212 (17)
Sulfonylurea	52,586 (3)	23,550 (5)	0 (0)	29,036 (6)
TZD	2,514 (<1)	1,238 (<1)	0 (0)	1,276 (<1)
DPP-4 inhibitor	11,309 (1)	4,841 (1)	0 (0)	6,468 (1)
GLP-1 receptor agonist	6,236 (<1)	2,746 (1)	0 (0)	3,490 (1)
SGLT-2 inhibitor	3,074 (<1)	1,177 (<1)	0 (0)	1,897 (<1)
**Cardiovascular risk**				
Statin	345,795 (18)	121,583 (24)	141,868 (15)	82,344 (16)
ASA	377,579 (19)	116,791 (23)	191,530 (20)	69,258 (14)
Other anti-platelet agent	44,532 (2)	15,527 (3)	18,879 (2)	10,126 (2)
**Nephrotoxins**				
NSAID	701,493 (36)	160,450 (32)	429,886 (45)	111,157 (22)
PPI	295,804 (15)	88,072 (17)	150,695 (16)	57,037 (11)

CKD-chronic kidney disease; CURE-CKD-Center for Kidney Disease Research, Education, and Hope; DM-diabetes mellitus; PDM-pre-diabetes mellitus; HTN-hypertension; N-overall sample size; n-prescription by group (non-mutually exclusive, i.e. participants may be on multiple/combination medications); ACE-angiotensin converting enzyme; ARB-angiotensin II receptor blocker; DRI-direct renin inhibitor; MRA-mineralocorticoid receptor antagonist; CCB-calcium channel blocker; TZD-thiazolidinedione; DPP-dipeptidyl peptidase; GLP-glucagon-like peptide; SGLT-sodium glucose co-transporter; ASA-acetylsalicylic acid; NSAID-nonsteroidal anti-inflammatory drug; PPI-proton pump inhibitor; [a]Other vasodilators-hydralazine and minoxidil

© 2019 Tuttle KR et al. *JAMA Network Open.*
